# Mechanically Strong, Freeze‐Resistant, and Ionically Conductive Organohydrogels for Flexible Strain Sensors and Batteries

**DOI:** 10.1002/advs.202206591

**Published:** 2023-01-19

**Authors:** Jiayu Lyu, Qingya Zhou, Haifeng Wang, Qi Xiao, Zhe Qiang, Xiaopeng Li, Jin Wen, Changhuai Ye, Meifang Zhu

**Affiliations:** ^1^ State Key Laboratory for Modification of Chemical Fibers and Polymer Materials College of Materials Science and Engineering Donghua University Shanghai 201620 China; ^2^ School of Polymer Science and Engineering The University of Southern Mississippi Hattiesburg MS 39406 USA

**Keywords:** aramid nanofibers, conductive hydrogels, freeze‐resistant, molecular dynamics simulations

## Abstract

Conductive hydrogels as promising material candidates for soft electronics have been rapidly developed in recent years. However, the low ionic conductivity, limited mechanical properties, and insufficient freeze‐resistance greatly limit their applications for flexible and wearable electronics. Herein, aramid nanofiber (ANF)‐reinforced poly(vinyl alcohol) (PVA) organohydrogels containing dimethyl sulfoxide (DMSO)/H_2_O mixed solvents with outstanding freeze‐resistance are fabricated through solution casting and 3D printing methods. The organohydrogels show both high tensile strength and toughness due to the synergistic effect of ANFs and DMSO in the system, which promotes PVA crystallization and intermolecular hydrogen bonding interactions between PVA molecules as well as ANFs and PVA, confirmed by a suite of characterization and molecular dynamics simulations. The organohydrogels also exhibit ultrahigh ionic conductivity, ranging from 1.1 to 34.3 S m^−1^ at −50 to 60 °C. Building on these excellent material properties, the organohydrogel‐based strain sensors and solid‐state zinc–air batteries (ZABs) are fabricated, which have a broad working temperature range. Particularly, the ZABs not only exhibit high specific capacity (262 mAh g^−1^) with ultra‐long cycling life (355 cycles, 118 h) even at −30 °C, but also can work properly under various deformation states, manifesting their great potential applications in soft robotics and wearable electronics.

## Graphical abstract

Organohydrogels simultaneously with high mechanical properties, ionic conductivity, and outstanding freeze resistance are prepared and used to fabricate flexible strain sensors and solid‐state zinc–air batteries (ZABs) with excellent comprehensive properties. Specifically, the organohydrogel‐based ZABs exhibited high energy capacity and ultra‐long cycling life both at room temperature and −30 °C.

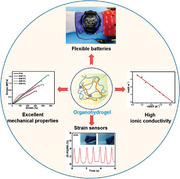

## 1 Introduction

Flexible conductive materials have attracted tremendous attention due to the rapid development of soft electronics in various applications, such as soft robotics and wearable electronics.^[^ ^1^, ^2^, ^3^, ^4^, ^5^ ^]^ Conductive hydrogels, which contain a large amount of water in polymer networks, are one of the most important material candidates for preparing soft electronics and devices due to their unique properties, including excellent mechanical flexibility, biocompatibility, and tunable ionic conductivity.^[^ ^6^, ^7^, ^8^ ^]^ In recent years, various hydrogel‐based systems have been demonstrated for preparing wearable electronics and devices, such as strain sensors and energy storage devices (flexible capacitors and batteries).^[^ ^9^, ^10^, ^11^, ^12^ ^]^ However, the poor mechanical properties of conventional hydrogels, such as low strength and brittleness, have significantly limited the practical use of these materials in many scenarios, where more challenging mechanics beyond stretchability, such as high tolerance in mechanical loading, bending, twisting, and folding are typically required.^[^ ^13^ ^]^ In addition, while the ionic conductivity can significantly impact the electrochemical performance of hydrogel‐based solid‐state batteries, the trade‐off between ionic conductivity and mechanical properties due to the influence of the cross‐linking density of polymer networks leads to the difficulty in fabricating hydrogel‐based energy storage devices with excellent comprehensive properties.^[^ ^14^, ^15^ ^]^

Significant efforts have been devoted to improving the mechanical properties of hydrogels, such as rational design of their chemical structure, control over materials morphology at multiple length scales, and introduction of reinforced nanoparticles.^[^ ^16^, ^17^, ^18^, ^19^ ^]^ For example, nanocomposite hydrogels were fabricated by the introduction of rigid nanoparticles into a soft polymeric matrix, which can exhibit significantly improved mechanical strength.^[^ ^20^ ^]^ However, conventional nanocomposite hydrogels typically show a trade‐off between mechanical strength and toughness, similar to most other polymer nanocomposites.^[^ ^6^ ^]^ The mechanical strength and toughness can be simultaneously improved by the construction of double‐network structures or by introducing additional reversible bonds in polymer networks.^[^ ^21^, ^22^ ^]^ For example, supramolecular double‐network hydrogels composed of permanent cross‐linked polyacrylamide network and self‐assembled octopeptide network showed high compressive modulus and toughness under the optimized polymer composition.^[^ ^23^ ^]^ Sun et al. synthesized tough hydrogels based on rationally designed polyampholytes containing ionic bonds with different strengths, in which the strong ionic bonds provided permanent cross‐linking sites while weak ionic bonds served as sacrificial bonds that dissipated mechanical energy during deformation.^[^ ^24^ ^]^ However, these approaches typically require sophisticated materials recipes or involve complicated synthesis.

Furthermore, the freezing of hydrogels at subzero temperatures is another important factor that hinders the implementation of hydrogel‐based soft electronics in a broad temperature range due to the relatively high freezing point of water. The freezing of water is primarily ascribed to the formation of a hydrogen bond network between water molecules, which can be inhibited by the introduction of polar organic solvents or salts. The addition of salts in water disrupts the usual network of hydrogen bonds made upon freezing, resulting in lowered freezing point of water.^[^ ^25^, ^26^ ^]^ Similarly, organic solvents, such as dimethyl sulfoxide (DMSO) and ethylene glycol, have been reported to improve the freeze‐resistance of organohydrogels through the strong hydrogen bonding interactions between organic solvents and water molecules.^[^ ^10^, ^27^, ^28^ ^]^ For example, conductive organohydrogels using DMSO as an anti‐freeze additive can exhibit a very high ionic conductivity of 1.1 S m^−1^ at −70 °C.^[^ ^18^ ^]^ However, the ratio of DMSO to water in organohydrogels may also impact the ionic conductivity and mechanical properties of organohydrogels, the mechanism of which has not been fully understood yet. Moreover, while low‐temperature tolerant organohydrogel‐based electronics, such as strain sensors and batteries, have been reported recently,^[^ ^29^, ^30^ ^]^ it still remains challenging to simultaneously achieve high electrical performance and excellent mechanical properties through a simple and potentially scalable fabrication strategy.

Herein, we demonstrate a simple method to fabricate ionically conductive aramid nanofiber (ANF) reinforced polyvinyl alcohol (ANF‐PVA) organohydrogels, which simultaneously exhibit very high ionic conductivity, excellent mechanical properties, and outstanding freeze‐resistance. The incorporation of a low amount of ANFs in the PVA network can significantly improve the crystallization of PVA during a freeze‐thaw process while the strong hydrogen bonding interactions between ANF molecules and PVA polymer chains can act as dynamic cross‐linking sites. Using molecular dynamics (MD) simulations, we confirm that DMSO in ANF‐PVA organohydrogels can significantly promote the intermolecular interactions between PVA polymer chains, thus improving the mechanical properties of organohydrogels while retaining high ionic conductivity. Moreover, the organohydrogels can exhibit ultrahigh ionic conductivity at a broad temperature range, which can be tuned by the DMSO to H_2_O ratio and salt concentration in the system. Building on these excellent properties, ANF‐PVA organohydrogel‐based strain sensors and solid‐state zinc–air batteries (ZABs) were fabricated with outstanding performance at a very broad range of operating temperatures, even down to −30 °C, demonstrating their great potential in the applications of flexible and wearable electronics.

## 2 Results and Discussion

### 2.1 Fabrication of ANF‐PVA Organohydrogels

**Figure 1:**
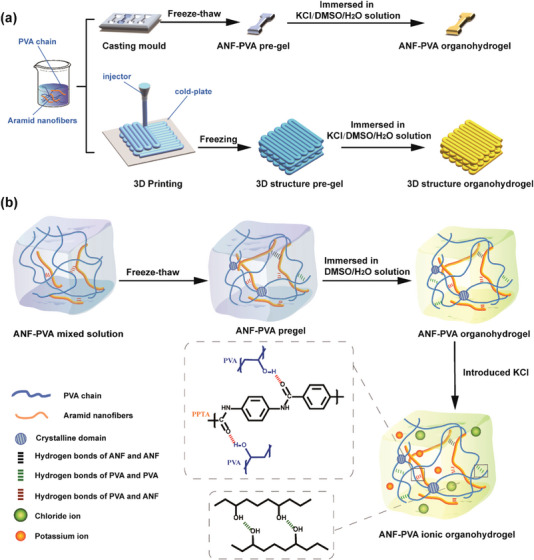
Schematic illustration of a) the preparation procedures of the ANF‐PVA organohydrogels by solution casting in a mold and 3D printing; b) the formation and microstructure of ANF‐PVA organohydrogels.

ANF‐PVA organohydrogels can be prepared either through solution casting using a mold or direct 3D printing, followed by a freezing‐thawing and solvent exchange process as shown in **Figure** **1**a. ANFs (1D nanofillers) were employed to enhance the mechanical properties of as‐prepared ANF‐PVA composite organohydrogels. ANFs with large slender ratios (diameter:7–25 nm, length: >10 µm, Figure S2, Supporting Information) were derived by dissolving commercial Kevlar threads in DMSO/KOH solution through a deprotonation reaction, which have ultrahigh mechanical properties similar to their macroscale‐parent Kevlar.^[^ ^6^, ^31^ ^]^ Additionally, amide groups on the ANF polymer chains are able to form strong hydrogen bonding interactions with hydroxyl groups from PVA molecules, which is beneficial for enhancing the mechanical properties of organohydrogels. The schematic illustration of the formation and microstructure of ANF‐PVA organohydrogel is shown in Figure 1b. ANFs can be uniformly dispersed in PVA organohydrogel (Figure S3, Supporting Information) by mixing ANF/DMSO and PVA/DMSO solution together followed by a freezing‐thawing process. The crystallization of PVA chains at low‐temperature results in the formation of PVA gel in DMSO. Since the breaking of crystalline domains of PVA in DMSO or H_2_O typically requires a temperature higher than 80 °C, these crystalline domains can act as permanent cross‐linking sites for organohydrogels. Hydrogen bonding, as a type of non‐covalent, secondary interaction with the dynamic and reversible nature, has been widely utilized to construct physically cross‐linked hydrogels.^[^ ^32^, ^33^, ^34^ ^]^ The hydrogen bonds in a hydrogel can serve as sacrificial bonds, which dissociate and re‐associate at different force levels to effectively dissipate mechanical energy and improve the toughness of hydrogels. In our system, the intermolecular hydrogen bondings between PVA and ANFs as well as hydrogen bonds between different PVA polymer chains provide dynamic cross‐linking sites by reversible breaking and reforming under stress, which can improve the mechanical properties of PVA organohydrogels. DMSO in ANF‐PVA organohydrogels has strong interactions with H_2_O, which affects the preferential solvation of PVA molecular chains by H_2_O. Since the gelation is induced through a freezing process, 3D printing can be employed to on‐demand prepare ANF‐PVA organohydrogels with controlled shapes by using a conventional extrusion‐based printer equipped with a cold stage. The introduction of K^+^ and OH^−^ ions in the system through a solvent exchange process using KOH/DMSO/H_2_O solution enables the organohydrogels with tunable ionic conductivity, which is promising for their applications in flexible electronics, such as strain sensors to monitor the motions of humans and flexible battery devices.

### 2.2 Effect of ANFs on the Mechanical Properties of ANF‐PVA Organohydrogels

**Figure 2:**
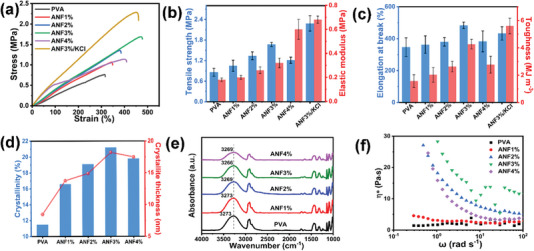
The mechanical properties of the ANF‐PVA organohydrogels with different ANF contents. a) Stress–strain curves of organohydrogels. b) Tensile stress and elastic modulus; c) elongation at break and toughness of organohydrogels with different contents of ANFs. d) The crystallinity and the crystal lamellae thickness; e) FTIR spectra; f) complex viscosity versus angular frequency of the PVA hydrogel and ANF‐PVA organohydrogels with increasing ANF content.

As ANFs can form strong hydrogen bonding interactions with PVA, the content of ANFs in the resulting organohydrogels significantly impacts their mechanical properties (**Figure** **2**). The solvent in organohydrogels was a mixture of DMSO/H_2_O (mass ratio of DMSO:H_2_O = 1:2) and the solvent fraction in all organohydrogels was ≈80% (Figure S1, Supporting Information). The elastic modulus of ANF‐PVA organohydrogels increases when adding a small amount of ANFs to the system. Specifically, the elastic modulus increases from 0.18 to 0.6 MPa as the ANF content increases to 4 wt%. In addition to the enhancement of elastic modulus with the addition of ANFs, the tensile strength of the ANF‐PVA organohydrogels is also significantly improved by introducing ANFs, slightly increasing from 0.86 to 1.05 MPa when adding 1 wt% of ANFs in ANF‐PVA organohydrogel, and reaching a maximum of 1.67 MPa when the ANF content is 3 wt%. This result corresponds to a 94% increase compared to that of PVA organohydrogel with the absence of ANFs. However, the tensile strength starts to decrease when further increasing the ANF content to 4 wt%, which is probably due to the aggregation of ANFs.

The enhancement of the mechanical strength and modulus of a composite induced by inorganic nanofillers typically leads to a decrease in toughness, which results in a reduction of the breaking strain.^[^ ^35^ ^]^ Interestingly, in our system the addition of ANFs not only enhances the tensile strength and modulus but also significantly improves the elongation at break of the organohydrogels. The elongation at break increases from 347% to 484% when ANF content increases from 0% to 3%. The elongation at break decreases when the ANF content further increases, which is consistent with tensile strength results. Correspondingly, the toughness of the organohydrogels is improved and reaches a maximum of 4.27 MJ cm^−3^ at ANF content of 3 wt%, which is 2.7 times that of neat PVA organohydrogel. The improvement of mechanical properties of ANF‐PVA organohydrogels is primarily due to the strong hydrogen bonding interactions between ANF and PVA as well as the ANFs inducing the enhanced crystallization of PVA. Interestingly, the mechanical properties of ANF‐PVA organohydrogel containing 3 wt% ANFs and 1.5 m KCl solution are superior compared to that of organohydrogel with the absence of salts probably due to the Hofmeister effect, which induces stronger and closer interphase interaction between PVA.^[^ ^17^, ^36^ ^]^

The crystalline domains of PVA polymer chains induced by a freezing process provide permanent cross‐linking sites, which can improve the mechanical properties of ANF‐PVA organohydrogels. The crystallinity and thickness of crystal lamellae of PVA in ANF‐PVA organohydrogels were characterized using differential scanning calorimetry (DSC) as shown in Figure S4, Supporting Information, and Figure 2d. The crystallinity of PVA in organohydrogel without the addition of ANFs is 11.5%. The addition of ANFs significantly enhances the crystallization of PVA polymer chains and a maximum crystallinity of 21.2% is achieved for organohydrogel with 3 wt% of ANFs (Figure 2d), indicating the ANFs can provide additional nucleation sites to effectively promote the crystallization of PVA polymer chains in ANF‐PVA organohydrogels, which results in significantly improved mechanical properties of the organohydrogels.

Additionally, while the melting temperature of PVA in organohydrogel without ANFs is 203.2 °C, the melting point increases to 224.6 °C for organohydrogel with 3 wt% ANF content. The melting temperature is related to the crystal lamellae thickness according to the Gibbs–Thomson equation:

(1) $$T_{m}^{\infty }-T_{m}=2T_{m}^{\infty }\frac{\gamma }{\Delta H_{V}^{\infty }}\frac{1}{l}$$

where *T*_m_ is the measured melting point, $T_{m}^{\infty }$ is the equilibrium melting temperature, which is 516.5 K for neat PVA.^[^ ^37^ ^]^ $\Delta H_{V}^{\infty }$, *l*, and *γ* are the melting enthalpy per unit volume for a crystal with infinite dimensions, crystal lamellae thickness, and solid–liquid interface energy, respectively. The value of 3.3 × 10^−8^ cm was used for $\gamma /\Delta H_{V}^{\infty }$ for PVA.^[^ ^38^ ^]^ The crystal lamellae thickness of PVA in ANF‐PVA organohydrogels is calculated and shown in Figure 2d. The lamellar crystal thickness significantly increases from 8.5 to 18.2 nm when the ANF content increases from 0 to 3 wt%. These results indicate that the introduction of a small amount of ANFs can significantly refine the crystallization of PVA and enhance the crystallinity, yielding significantly improved mechanical properties of ANF‐PVA organohydrogels.

Furthermore, the strong interactions between ANFs and PVA chains due to the hydrogen bonding interactions can also provide dynamic cross‐linking sites for the ANF‐PVA organohydrogels. These dynamic cross‐linking sites can act as “sacrificial domains” for mechanical energy dissipation during the stretching of the organohydrogels, which also contributes to the mechanical property enhancement. The hydrogen bonding interactions between ANFs and PVA chains were revealed by Fourier transform infrared spectroscopy (FTIR) as shown in Figure 2e. The peak at 3273 cm^−1^ corresponds to stretching vibrations of hydroxyl groups (O—H) in PVA.^[^ ^39^ ^]^ A redshift of stretching vibrations of hydroxyl groups was observed when adding ANFs in the organohydrogels, confirming the hydrogen bonding interactions between ANF and PVA.^[^ ^40^ ^]^ Additionally, rheological tests were conducted to further reveal the hydrogen bonding interactions between ANFs and PVA molecular chains (Figure 2f). The complex viscosity at low frequency (<1 rad s^−1^) of ANF‐PVA solution with the addition of >2 wt% ANF is more than one magnitude higher than that of PVA solution, indicating significantly increased intermolecular attractions caused by hydrogen bonding interactions between ANFs and PVA, which hinders the movements of PVA polymer chains. Correspondingly, the storage modulus and loss modulus show a similarly growing trend when adding a small amount of ANFs in PVA solutions (Figure S5, Supporting Information), further confirming the strong hydrogen bonding interactions between ANFs and PVA, which results in an improvement of mechanical properties for ANF‐PVA organohydrogels.

### 2.3 Effect of DMSO on the Mechanical Properties of ANF‐PVA Organohydrogels

**Figure 3:**
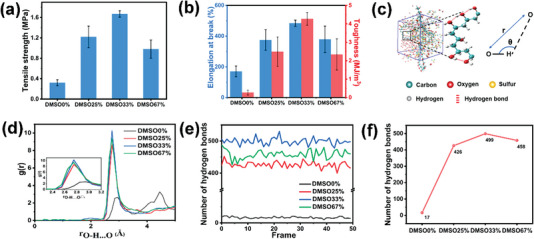
a) Tensile strength, b) elongation at break and toughness of ANF3%‐PVA organohydrogels with different DMSO content. c) Molecular modeling of PVA organohydrogel. d) Radial distribution function of oxygen atoms in PVA molecules, e) change in the number of hydrogen bonds, and f) the average number of hydrogen bonds formed between PVA molecules in organohydrogels with different DMSO contents.

DMSO as a widely used cryoprotectant for cryopreserving cells was recently reported for the fabrication of freeze‐resistant organohydrogels.^[^ ^18^, ^28^ ^]^ DMSO and H_2_O can form strong interactions in DMSO/H_2_O mixture, where two water molecules are present to hydrogen bond to every DMSO oxygen, resulting in non‐ideal behavior of the mixed solvent, such as extremely low freezing point.^[^ ^41^ ^]^ The strong interaction between DMSO and H_2_O is also supposed to affect the solvation of PVA chains by H_2_O molecules, thus affecting the mechanical properties of ANF‐PVA organohydrogels. The effect of DMSO to H_2_O ratios on the mechanical properties of ANF3%‐PVA organohydrogels was investigated and the results are shown in **Figure** **3** and Figure S6, Supporting Information. The tensile strength and toughness of ANF‐PVA organohydrogels are all higher than those of PVA hydrogels. The organohydrogel with a DMSO fraction of 33 wt% shows the highest tensile strength and toughness. This is because the solvation of PVA chains by free H_2_O molecules was suppressed due to the presence of DMSO, allowing the formation of stronger hydrogen bonding interactions between DMSO and H_2_O. Therefore, the intramolecular and intermolecular hydrogen bonding interactions between PVA polymer chains are enhanced, which leads to simultaneously improved mechanical strength and toughness of the organohydrogels. The high tensile strength and toughness achieved for the ANF‐PVA organohydrogels are higher than most reported hydrogel materials (Figure S7, Supporting Information).

MD simulations were conducted to verify the enhanced hydrogen bonding interactions between PVA polymer chains caused by the introduction of DMSO, through evaluating the number of hydrogen bonds between PVA polymer chains in DMSO/H_2_O mixed solvents with different fractions of DMSO. To simplify the simulated system, the structure of PVA organohydrogel was constructed in a simulation box with a periodic boundary condition, which includes 10 PVA polymer chains, DMSO, and H_2_O molecules as shown in Figure 3c. The hydroxyl groups’ distribution in PVA polymer chains can be estimated by using radial distribution functions, *g*_O—H…O_(*r*), which gives the local density of atoms *O* in hydroxyl groups of PVA around central atoms *O* of PVA at a distance of *r*, divided by the average density of the whole system.^[^ ^42^ ^]^ The formation of intermolecular hydrogen bonds in PVA polymer chains reduces the distance between hydroxyl groups in PVA, which can be evaluated by the radial distribution function *g*_O—H…O_(*r*). Figure 3d shows the effect of DMSO content in mixed DMSO/H_2_O solvents on the radial distribution function between hydroxyl groups *g*_O‒H…O_(*r*) in PVA polymer chains. A peak is observed for radial distribution function at an *r*_—OH···O_ value of 2.93 Å for PVA hydrogel with the absence of DMSO, suggesting a hydrogen bond regime in the hydration shell of PVA hydroxylic groups. The distance of *r*_—OH···O_ at the maximum *g*_O‒H…O_(*r*) reduces to 2.75 Å as the DMSO content in DMSO/H_2_O increases to 33 wt%, suggesting that the addition of DMSO suppresses the solvation of PVA chains by H_2_O and promotes the formation of intermolecular hydrogen bonds in PVA, leading to the reduced average distance between hydroxyl groups in PVA polymer chains.

The hydrogen bonds between PVA hydroxylic groups were further investigated by analyzing the trajectory for the occurrence of this interaction, adopting as geometric criteria an acceptor–donor distance (—OH···O) smaller than 0.3 nm and an angle *θ* lower than 180°. The average number of hydrogen bonds in PVA organohydrogels containing DMSO is all larger than 400, which increases by more than 25 times compared to that of PVA hydrogels without the addition of DMSO (Figure 3e,f). The average number of hydrogen bonds between PVA polymer chains reaches a maximum for organohydrogel with 33% DMSO in the system, which is consistent with the results of mechanical properties of the ANF‐PVA organohydrogels (Figure 3a).

### 2.4 Ionic Conductivity and Anti‐Freezing Properties of the ANF‐PVA Organohydrogel

**Figure 4:**
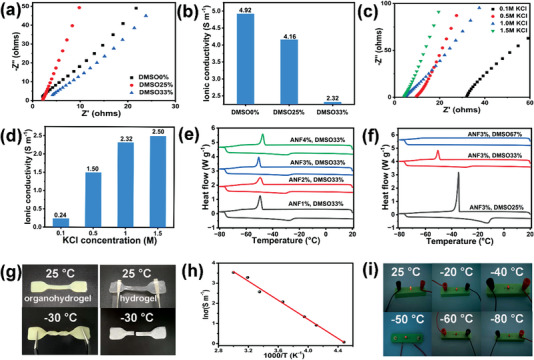
a) Electrochemical impedance spectra, and b) ionic conductivity of ANF‐PVA organohydrogels with different DMSO contents using 1 m KCl for solvent exchange. c) Electrochemical impedance spectra, and d) ionic conductivity of ANF‐PVA organohydrogels with 33% DMSO using different concentrations of KCl solutions for solvent exchange. DSC curves for organohydrogels with different e) ANF contents (33 wt% DMSO) and f) DMSO contents (3 wt% ANFs). g) Freeze‐resistance of PVA hydrogel and ANF‐PVA‐DMSO33% organohydrogel. h) Temperature‐dependent conductivity of organohydrogel containing 6 m KOH. i) Performance of the organohydrogel as a conductor under different temperatures.

The ANF‐PVA organohydrogels exhibit tunable ionic conductivity by introducing different amounts of salts, such as KCl and KOH, into the gels through a simple solution‐soaking process. The ionic conductivity of the organohydrogels was characterized by electrochemical impedance spectroscopy and the results are shown in **Figure** **4**a–d. The ionic conductivity of organohydrogels (soaking in 1.0 m KCl) decreases when the DMSO content in H_2_O/DMSO‐mixed solvents increases from 0 to 33 wt% due to the suppressed ion dissociation of salts in DMSO. However, the conductivity of the organohydrogel can still reach 2.32 S m^−1^ when the DMSO content is 33 wt% due to the relatively higher dielectric constant of DMSO compared to other anti‐freezing organic additives, such as ethylene glycol.^[^ ^43^ ^]^ The effect of KCl concentration on the conductivity of organohydrogels was investigated as shown in Figure 4c,d. The conductivity gradually increases from 0.24 to 2.5 S m^−1^ when the KCl concentration increases from 0.1 to 1.5 m. The organohydrogels prepared using KOH as the soaking solution also show outstanding ionic conductivity. Particularly, an extremely high ionic conductivity of 13.1 S m^−1^ at 25 °C is achieved for organohydrogels using 6 m KOH as the soaking solution, which is higher than most previously reported organohydrogels.^[^ ^44^, ^45^, ^46^ ^]^ The high ionic conductivity of the organohydrogels is attributed to the high dielectric constant of the added DMSO, which is beneficial for the ion dissociation of salts compared to those organic solvents with a lower dielectric constant. Additionally, since the polymer chains could have a blocking effect on ion transport, the high solvent content in the organohydrogels results in a relatively lower density of polymer chains per unit volume, which can further enhance the ion transport in the organohydrogels. The high and tunable ionic conductivity can enable the ANF‐PVA organohydrogels to be an excellent material candidate for flexible electronics with exceptional performance.

Moreover, DMSO as a widely used cryoprotectant for living cells can reduce the freezing point of H_2_O, which endows the ANF‐PVA organohydrogels with excellent anti‐freezing properties. The initial freezing point of organohydrogels (33 wt% DMSO in H_2_O/DMSO binary solvent) with different ANF content ranging from 1 to 4 wt% is −47 ± 2 °C as determined by DSC (Figure 4e and Table S1, Supporting Information), indicating that the freezing of organohydrogels is almost not affected by the addition of ANFs. However, the initial freezing temperature of the organohydrogels is significantly lower compared to that of PVA hydrogels (≈−13 °C, Figure S8, Supporting Information). The significant suppressed freezing point is attributed to the formation of DMSO/H_2_O clusters due to the strong hydrogen bonding interactions between DMSO and H_2_O molecules, which disrupts the hydrogen bonding network of water and inhibits the formation of ice crystals.^[^ ^18^, ^47^ ^]^ The fraction of DMSO in the DMSO/H_2_O mixture also impacts the freezing point of organohydrogels (Figure 4f). The freezing temperature significantly lowered when using a higher content of DMSO. The freezing of the mixed solvents in organohydrogels occurs at a broad temperature range when the content of DMSO is 67 wt%, which is difficult to detect by DSC. The organohydrogel maintains excellent mechanical flexibility, which can be twisted even at very low temperatures, such as −30 °C, (Figure 4g) indicating its excellent anti‐freezing properties. As a comparison, the transparent PVA hydrogel becomes opaque and can be easily fractured upon bending at −30 °C due to the freezing of water in the hydrogel. The temperature‐dependent ionic conductivity of the ANF‐PVA organohydrogels containing 6 m KOH is determined from Nyquist plots (Figure S9, Supporting Information), which follows Arrhenius relation as *σ* = *σ*_0_ exp(−*E*_a_/*RT*) at the examined temperature ranging from −50 to 60 °C as shown in Figure 4h, where *σ*_0_ is a pre‐exponential factor and *E*_a_ is the activation energy. This indicates the ion transport in the organohydrogel is thermally activated and the activation energy is 19.7 kJ mol^−1^ as determined from the Arrhenius plot. While the ionic conductivity of organohydrogels decreases from 34.3 to 1.1 S m^−1^ as the temperature decreases from 60 to −50 °C due to the decreased mobility of the ions with the temperature, the organohydrogels (6 m KOH) still exhibit high conductivity even at −50 °C, due to the significantly depressed freezing point (Figure 4h). The LED lamp can be lit using the organohydrogel as the conductor at a temperature above −50 °C (Figure 4i), further confirming the high ionic conductivity retained above the freezing point of the organohydrogels.

### 2.5 3D Printing and Application of Organohydrogels for Strain Sensing

**Figure 5:**
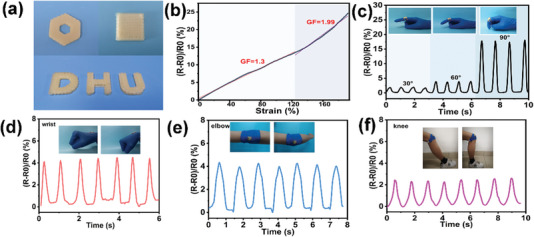
a) Photographs of 3D‐printed ANF‐PVA organohydrogels with the designed shapes. b) The relative resistance change of the 3D‐printed organohydrogel at different stretching strains. Conductive organohydrogels as strain sensors to monitor the movements of bending of c) a finger, d) wrist, e) elbow, and f) knee.

The simple preparation method using a freeze‐thaw process enables the fabrication of the ANF‐PVA organohydrogels using 3D printing method with complex and designed shapes, which are especially important for the applications of soft robotics. The ANF‐PVA organohydrogels with designed shapes (hollow hexagon, quadrilateral, and letters of *D*, *H*, and *U*) were prepared by 3D printing using a cold stage setting at −20 °C (**Figure** **5**a), which can rapidly freeze the printed patterns of the organohydrogels. The high flexibility, tunable conductivity, and programmable shape enable organohydrogels as strain sensors for detecting the movements of humans. The sensing properties of the 3D‐printed organohydrogels were investigated and shown in Figure 5b. A linear response of the relative resistance change to stretching strain was observed at the strain range between 0% and 125%. The sensitivity of the strain sensor which is evaluated using GF is 1.3 at a broad strain range. The GF increases to 1.99 at the stretching strain larger than 125%. The sensitivity of the strain sensor using the 3D printed organohydrogel is comparable with or higher than most previously reported strain sensors based on ionically conductive hydrogels or organohydrogels (Figure S10, Supporting Information). Moreover, it was demonstrated that the strain sensors based on the as‐prepared organohydrogels can detect various human activities, such as bending the fingers, wrist, elbow, and knees, as shown in Figure 5c‐f.

### 2.6 Application of Organohydrogels for Flexible Battery

**Figure 6:**
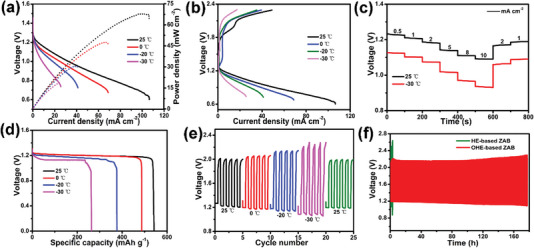
Electrochemical performance of ANF‐PVA organohydrogel‐based ZABs at different temperatures. a) Discharge and power density curves. b) Charge/discharge plots. c) Discharge profiles at different current densities. d) Specific discharge capacity plots recorded at the current density of 2 mA cm^−2^. e) Cycling performance at a current density of 1 mA cm^−2^ at different temperatures. f) Cycling performance at a current density of 1 mA cm^−2^ at −20 °C.

With the rapid development of flexible electronics, ZABs have attracted much attention due to the increasing demand for power supply devices with thin, flexible, and even stretchable. However, traditional ZABs using aqueous electrolytes have intrinsic disadvantages of liquid leakage, narrow working temperature window, and low mechanical flexibility, which cannot meet the requirement of the power supply for flexible and wearable electronics. The exceptional mechanical properties, outstanding anti‐freezing properties, and high ionic conductivity enable the ANF‐PVA organohydrogels to be employed for fabricating flexible ZABs with a very broad range of operating temperatures. Specifically, flexible solid‐state ZABs were fabricated using ANF‐PVA organohydrogel (6 m KOH) as the electrolyte to further demonstrate the practical use of these organohydrogels in energy applications. The polarization curve and output power of the flexible ZABs at different temperatures are shown in **Figure** **6**a. The maximum output power of ZAB reaches 67.5 mW cm^−2^ at room temperature and reduces when the temperature decreases, which is due to the reduced ion conductivity of the ANF‐PVA organohydrogel electrolyte (OHE) at lower temperatures. However, the maximum output power still reaches 17.9 mW cm^−2^ when the ZAB is operated at a very low temperature of −30 °C, which is critical for the use of flexible ZABs in cold environments. The organohydrogel‐based ZABs can be operated at different current densities and temperatures as low as −30 °C (Figure 6b). The discharge step diagrams of the flexible ZABs at 25 and −30 °C are shown in Figure 6c. While the ZABs at room temperature and −30 °C both deliver a stable voltage output, the discharge voltage of the ZABs at different current densities at 25 °C is all higher than that of ZABs at −30 °C. The discharge performance of the ZABs decreases as the current density increases. However, the decrease in discharge plateaus is only 0.2 V when the current density increases from 0.5 to 10 mA cm^−2^. More importantly, the ZAB can still work at the current density of 10 mA cm^−2^ even at the temperature of −30 °C due to the high ionic conductivity of ANF‐PVA organohydrogels at low temperatures, indicating the excellent freeze‐resistance of the organohydrogel‐based ZABs. ZABs achieve a high specific capacity of 542 mAh g^−1^ at the current density of 2 mA cm^−2^, which corresponds to an energy density and areal capacities of 650 mWh g^−1^ and 28 mAh cm^−2^, respectively (Figures S11 and S12, Supporting Information). The specific capacity can reach 262 mAh g^−1^ at a subzero temperature of −30 °C (Figure 6d), demonstrating high capacity retention at low temperatures. Moreover, the ANF‐PVA organohydrogel‐based ZABs exhibit high cycle stability at a broad temperature range from room temperature to subzero temperature as shown in Figure 6e.

The long‐term cycling tests with a duration of 20 min per cycle were performed for ANF‐PVA organohydrogel‐based ZABs and PVA hydrogel‐based ZABs, as shown in Figure 6f. The performance of the organohydrogel‐based ZAB is stable for more than 42 h (127 cycles) at room temperature for the cycling test, indicating excellent cycling stability of the battery. Interestingly, the cycle stability of the organohydrogel‐based ZABs improves at lower temperatures (Figures S13–S15, Supporting Information). And ultrahigh long‐term stability (>177 h, over 532 cycles) is achieved at the optimal temperature of −20 °C, which is more than four times better than that at room temperature and much higher than that of PVA hydrogel‐based ZABs (3 h, 9 cycles). The ZABs can still remain high cycling stability up to 118 h (355 cycles) even at −30 °C, indicating the outstanding performance of the organohydrogel‐based ZABs at subzero temperatures. The superior long‐term performance of organohydrogel‐based ZABs is probably attributed to the suppressed corrosion of the zinc anode and the side reaction of the hydrogen evolution reaction.

**Figure 7:**
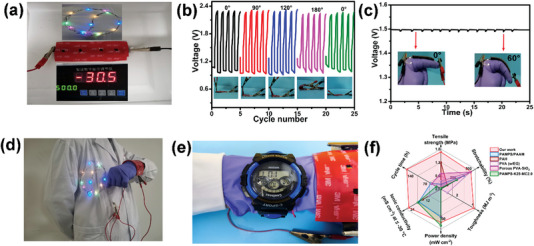
a) LED belt working at −30 °C using the ANF‐PVA organohydrogel‐based ZAB as the power supply. b) Cycling stability of the ZAB under different deformations at −30 °C. c) Open circuit voltage change of the ZAB under deformation. Demonstrations of the ZAB as a wearable battery for d) LED belt and e) watch. f) A comprehensive comparison between ANF‐PVA organohydrogels of this work and previously reported conductive hydrogels in terms of tensile strength, stretchability, toughness, and ionic conductivity at ≤−30 °C, as well as power density and cycling stability (≤−20 °C) as ZABs.^[^ ^28^, ^48^, ^49^, ^50^, ^51^ ^]^

The high mechanical flexibility and low‐temperature tolerance enable the ANF‐PVA organohydrogel‐based ZABs as the wearable power supply for their applications in soft robotics. The ZAB can continuously supply electrical power for a LED belt containing 20 bulbs even at −30 °C (**Figure** **7**a). Moreover, the charge/discharge curves are almost the same for the ZABs upon bending at different angles from 0° to 180° at both room temperature and −30 °C (Figure 7b and Figure S16, Supporting Information), indicating the excellent performance stability of the ZABs under a large degree of deformation and at a broad temperature range. The ZAB adhered to a finger shows a stable open circuit voltage of ≈1.5 V as shown in Figure 7c. The maximum voltage change of the ZAB is only 0.009 V when the ZAB attached to the finger repeatedly bends to an angle of 60°, demonstrating the feasibility of the flexible ZABs in the application of soft robotic, which requires a stable output voltage of the battery at various motions. The ZABs as flexible and wearable batteries for supplying power to common electronics, such as LED bulbs and watches, under the large scale of deformations are further demonstrated in Figure 7d,e and Figure S17, Supporting Information. The watch can work properly when the ZAB adhered to the skin of human continuously bends (Video S1, Supporting Information). Notably, the organohydrogel‐based ZABs show superior comprehensive performance among mechanical strength, toughness, stretchability, ionic conductivity, and cycling stability, which outperforms the vast majority of conductive hydrogels as flexible energy storage devices (Figure 7f), demonstrating the potential application of such conductive organohydrogels in wearable electronics and soft robotics.^[^ ^28^, ^48^, ^49^, ^50^, ^51^ ^]^

## 3 Conclusion

In summary, ANF‐PVA organohydrogels fabricated by solution casting and/or 3D printing can simultaneously exhibit excellent mechanical properties, ionic conductivity, and freeze resistance. The tensile strength and toughness of the organohydrogels were significantly improved due to the synergistic effect of ANFs and DMSO, which promoted the crystallization of PVA and hydrogen bonding interactions between PVA polymer chains as well as PVA and ANFs. The ionic conductivity of the organohydrogels was tuned by changing the ratios of DMSO/H_2_O and the concentration of the salt solution. High conductivity between 1.1 and 34.3 S m^−1^ was achieved at a broad temperature range down to −50 °C due to the excellent anti‐freezing properties induced by DMSO. Conductive organohydrogel‐based strain sensors were fabricated, which can be used to detect various human movements with high sensitivity. Moreover, the organohydrogel‐based ZABs can exhibit high output power and ultrahigh long‐term cycle stability at a broad temperature range, from room temperature to −30 °C. The ZABs also showed stable output power for various portable electronics under large deformation, demonstrating the feasibility of the organohydrogels for applications in flexible and wearable electronics. Notably, the strategy for preparing high‐performance organohydrogels is versatile and can be adapted to various hydrogel systems, which is promising for further extending the applications of organohydrogels in soft electronics.

## 4 Experimental Section

### Materials

PVA (Mw ≈ 145 000 Da, 98% hydrolysis) was purchased from Meryer Co., Ltd. Kevlar fibers were purchased from Shanghai Branch, DuPont China Holding Co., Ltd. Potassium hydroxide (KOH, AR) and potassium chloride (KCl, AR) were purchased from Sinopharm Chemical Reagent Co., Ltd. DMSO (AR) was purchased from Shanghai Titan Scientific Co., Ltd. Zinc foil (thickness: 0.08 mm) was provided by Institute of High Purity Metal Materials. Carbon nanotube (CNT) was purchased from Nanjing XFNANO Materials Tech Co., Ltd. Carbon cloth (W1S1011, 0.41 mm thick) was supplied by CeTech Co., Ltd. All reagents were used without further purification.

### Fabrication of Aramid Nanofiber‐Reinforced PVA Organohydrogels and Electrolytes

2 wt% ANF solution was prepared by mixing 1.8 g Kevlar, 2.7 g KOH, and 85.5 g DMSO in a serum bottle and stirred at 65 °C for 4 days. 10 wt% PVA solution was prepared by adding 10 g PVA powder to 90 g DMSO with vigorous stirring at 90 °C for 3 h. ANF‐PVA solution with ANF weight fraction ranging from 1 to 4 wt% was prepared by mixing 2 wt% ANF solution and 10 wt% PVA solution at desired mass ratios at 60 °C under vigorous stirring. The ANF‐PVA solution was poured into a Teflon mold after removing bubbles under vacuum, and then kept at −45 °C for 10 h for gelation. Subsequently, the freezing ANF‐PVA gel was thawed at room temperature for 1 h. The freeze‐thaw process was repeated three times. ANF‐PVA hydrogels were obtained by soaking the as‐prepared gels in deionized H_2_O for 24 h, while ANF‐PVA organohydrogels were obtained by soaking the as‐prepared gels in DMSO/H_2_O mixed solvents for 24 h for completely solvent exchange. Similarly, the ANF‐PVA hydrogel electrolytes and OHEs were fabricated by immersing the gels in KCl or KOH aqueous solutions and KCl/DMSO/H_2_O or KOH/DMSO/H_2_O solutions for 24 h, respectively.

### Fabrication of Flexible Zinc–Air Batteries

Sandwich‐structured ZABs were fabricated using ionically conductive ANF‐PVA organohydrogels as the electrolyte, Zn foil as the anode, and Co/CNT catalyst‐coated carbon cloth as the cathode. The ANF‐PVA OHEs were obtained by soaking the gels in 6 m KOH/DMSO/H_2_O solution for 24 h. The Co/CNT catalyst was prepared according to the previously reported method.^[^ ^52^ ^]^ The carbon cloth was uniformly coated with Co/CNT catalyst with a loading of 0.4 mg cm^−2^.

### 3D Printing of the ANF‐PVA Organohydrogel

3D printing of organohydrogel was performed using a pneumatic device (GeSim bioscaffold printer) equipped with a cold platform. The ANF‐PVA/DMSO solutions were used as inks for 3D printing. The inks were squeezed out from a 0.6 mm microneedle and were immediately frozen into a designed shape on a cold platform. The 3D organohydrogel was obtained by keeping the frozen sample at −45 °C for 1 day and then immersing it in a solution of KCl/DMSO/H_2_O for solvent exchange.

### Simulation Details

MD simulations were performed to understand the hydrogen bonding interactions between PVA polymer chains in DMSO/H_2_O mixed solvents. A 200 Å × 200 Å × 200 Å box with the periodic boundary condition was created using the amorphous cell module in Materials Studio 6.0 to simulate the structures of PVA hydrogels and organohydrogels,^[^ ^53^ ^]^ which includes ten PVA chains (30 repeat units per chain), DMSO, and H_2_O molecules. DMSO and water molecules were created by Device Studio with the charge population calculated at the B3LYP/cc‐pVDZ level using Gaussian 16 version C.02.^[^ ^54^ ^]^ The molecule ratios of DMSO/H_2_O in the simulation box were tuned, resulting in different weight fractions of DMSO in DMSO/H_2_O mixture in the organohydrogel system. The Discover module in the Materials Studio 6.0 software package was used for geometry optimization and MD simulations. The polymer consistent force‐field was assigned on the network structure of PVA hydrogel during the geometry optimization by the steepest descent method.^[^ ^55^ ^]^ To reach the equilibrium state of the whole system, MD simulations were performed in an NVT ensemble with constant mole number (N), constant volume (V), and constant temperature (T), followed by simulations in an NPT ensemble with constant pressure (P) and constant temperature (T). Both NVT and NPT simulations were carried out in 200 ps with the time step of 1.0 fs in the equilibrium step. The temperature was set at 233 K controlled by Nose during NVT simulations,^[^ ^56^ ^]^ while the pressure was set at 1 GPa controlled by Berendsen for the NPT simulations.^[^ ^57^ ^]^ After the hydrogel network reached the equilibrium state, the NPT ensemble was carried out in 1 ns in the production process, during which the trajectories of the first 50 frames were selected. The number of hydrogen bonds was obtained by analysis function in VMD version 1.9 in 50 trajectories, where the hydrogen bond length and angle were lower than 3 Å and 180°, respectively.^[^ ^58^ ^]^

### Characterization

The chemical composition of the ANF‐PVA organohydrogel was characterized by FTIR (Thermo Nicolet NEXUS 670 spectrometer) using reflection mode with a wavenumber ranging from 4000 to 512 cm^−1^. Rheological measurements of the ANF‐PVA solution were conducted on HAAKE stress (German HAAKE company) with a frequency range of 0.01–100 Hz at 25 °C. The crystallinity of ANF‐PVA organohydrogels was measured using DSC (METTLER TOLEDO) by heating the samples from −20 to 300 °C at a rate of 5 °C min^−1^. Before DSC measurements, ANF‐PVA organohydrogels were immersed in excess glutaraldehyde hydrochloric for cross‐linking of amorphous polymer chains in PVA to avoid the crystallization of the amorphous chain during DSC measurements. Crystallinity of PVA in organohydrogels was calculated according to the following equation:

(2) $$X=\frac{H_{\mathrm{crystalline}}}{H_{\mathrm{crystalline}}^{0}}$$

where *H*_crystalline_ was the enthalpy for melting the crystalline domains per unit mass of the dry sample, and $H_{\mathrm{crystalline}}^{0}$ (138.6 J g^−1^) was the enthalpy of fusion of 100 wt% crystalline PVA.^[^ ^59^ ^]^ Since the melting temperature of ANFs was higher than the testing temperature range in this work, the enthalpy of melting that appeared in the DSC curves was attributed to the melting of crystalline domains of PVA in the organohydrogels. The mechanical properties of ANF‐PVA organohydrogel were measured using an electronic universal material testing machine (Instron 5969) with a strain rate of 50 mm min^−1^.

The strain sensing performance was evaluated by measuring the electrical resistance change of organohydrogels upon deformation. The resistance of organohydrogels was recorded using a digital multimeter (Keithley, DMM6500). Relative resistance (*R*_r_) was calculated as follows:

(3) $$R_{r}=\frac{\left(R-R_{0}\right)}{R_{0}}\times 100\%$$

where *R*_0_ and *R* were the original and real‐time resistances of the organohydrogel. Gauge factor (GF) of the organohydrogel sensors was calculated as GF = ∂*R*_r_/∂*ε*, where *ε* represents the strain.

The anti‐freezing property of the ANF‐PVA organohydrogel was studied using DSC. The prepared organohydrogels were cooled from 20 to −60 °C and then heated to 20 °C at a rate of −5 °C min^−1^. The ionic conductivity of PVA organohydrogels was measured by electrochemical impendence spectroscopy using an electrochemical workstation using a frequency range from 10^5^ to 10^−1^ Hz. The ionic conductivity of organohydrogels was determined according to the following equation:

(4) $$\sigma =\frac{L}{RA}$$

where *L* represented the thickness of organohydrogel, *R* represented the bulk resistance (the intercept at the real part in Nyquist plots), and *A* represented the area of the electrolyte. The electrochemical analyses of ZABs were performed using Princeton electrochemical workstation using a scan rate of 10 mV s^−1^. Discharging and cycling tests were performed on a multichannel battery testing system (LAND CT3002A) using a current density of 2 and 1 mA cm^−1^ with 20 min per cycle, respectively. The specific capacity (mAh g^−1^) and the energy density (mWh g^−1^) of ZABs were determined according to the following equations.

(5) $$\mathrm{Specific}\;\mathrm{capacity}=\frac{\mathrm{Discharge}\;\mathrm{current}\times \mathrm{Service}\;\mathrm{time}}{\mathrm{Mass}\;\mathrm{of}\;\mathrm{consumed}\;\mathrm{zinc}}$$

(6) $$\mathrm{Energy}\;\mathrm{density}=\frac{\begin{array}{c} \mathrm{Discharge}\;\mathrm{current}\times \mathrm{Average}\;\mathrm{discharge}\;\mathrm{voltage}\;\\ \times \mathrm{Service}\;\mathrm{time} \end{array}}{\;\mathrm{Mass}\;\mathrm{of}\;\mathrm{consumed}\;\mathrm{zinc}}$$

(7) $$\mathrm{Areal}\;\mathrm{specific}\;\mathrm{capacity}=\frac{\mathrm{Discharge}\;\mathrm{current}\times \mathrm{Service}\;\mathrm{time}}{\mathrm{Electrode}\;\mathrm{area}}$$

(8) $$\begin{array}{c} \mathrm{Areal}\;\mathrm{energy}\;\mathrm{density}=\frac{\begin{array}{c} \mathrm{Discharge}\;\mathrm{current}\;\times \;\mathrm{Average}\;\mathrm{discharge}\;\mathrm{voltage}\\ \times \;\mathrm{Service}\;\mathrm{time} \end{array}}{\;\mathrm{Electrode}\;\mathrm{area}} \end{array}$$

## Conflict of Interest

The authors declare no conflict of interest.

## Author Contributions

J.L. and Q.Z. contributed equally to this work. The manuscript was written through contributions of all authors. All authors have given approval to the final version of the manuscript.

## Supporting information

Supporting Information

Supplemental Movie 1

## Data Availability

The data that support the findings of this study are available from the corresponding author upon reasonable request.
