# Supplementary material for: Mechanically Strong, Freeze‐Resistant, and Ionically Conductive Organohydrogels for Flexible Strain Sensors and Batteries
Source: Adv Sci (Weinh). 2023 Jan 19;10(9):2206591. doi: 10.1002/advs.202206591 (PMC10037987; doi:10.1002/advs.202206591)
Supplement: Supplementary file 1 — Supporting Information [file ADVS-10-2206591-s001.pdf]

ADVANCED SCIENCE
Open Access

Supporting Information

for *Adv. Sci.*, DOI 10.1002/advs.202206591

Mechanically Strong, Freeze-Resistant, and Ionically Conductive Organohydrogels for Flexible Strain Sensors and Batteries

Jiayu Lyu, Qingya Zhou, Haifeng Wang, Qi Xiao, Zhe Qiang, Xiaopeng Li, Jin Wen, Changhuai Ye* and Meifang Zhu*

Supporting information for:

Mechanically strong, freeze-resistant, and ionically conductive organohydrogels for flexible strain sensors and batteries

Jiayu Lyu,[1]*# Qingya Zhou,*[1]*# Haifeng Wang,*[1] *Qi Xiao,*[1] *Zhe Qiang,*[2] *Xiaopeng Li,*[1] *Jin Wen,*[1]** Changhuai Ye,*[1]** Meifang Zhu*[1]

[1]State Key Laboratory for Modification of Chemical Fibers and Polymer Materials, College of Materials Science and Engineering, Donghua University, Shanghai 201620, China

[2]School of Polymer Science and Engineering, The University of Southern Mississippi, Hattiesburg, MS 39406, United States

[*]Corresponding author. E-mail addresses: jinwen@dhu.edu.cn (J. Wen) and cye@dhu.edu.cn (CH. Ye)

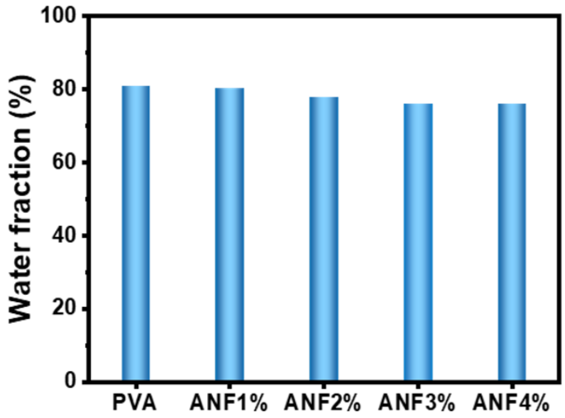

Figure S1. Water fraction of ANF-PVA organohydrogels with different ANF contents.

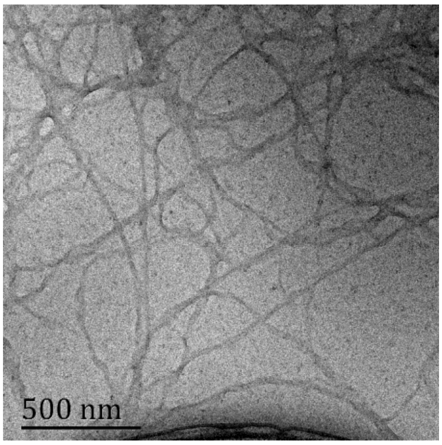

Figure S2. TEM micrograph of aramid nanofibers.

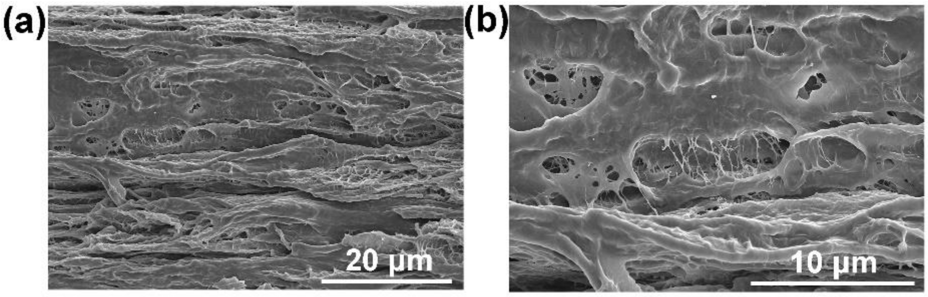

Figure S3. Cross-sectional SEM micrographs of ANF3%-PVA organohydrogels.

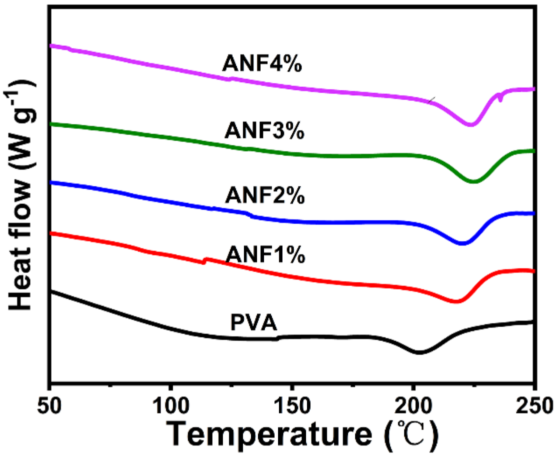

Figure S4. DSC curve of of the PVA hydrogel and ANF-PVA organohydrogels with increasing ANF content.

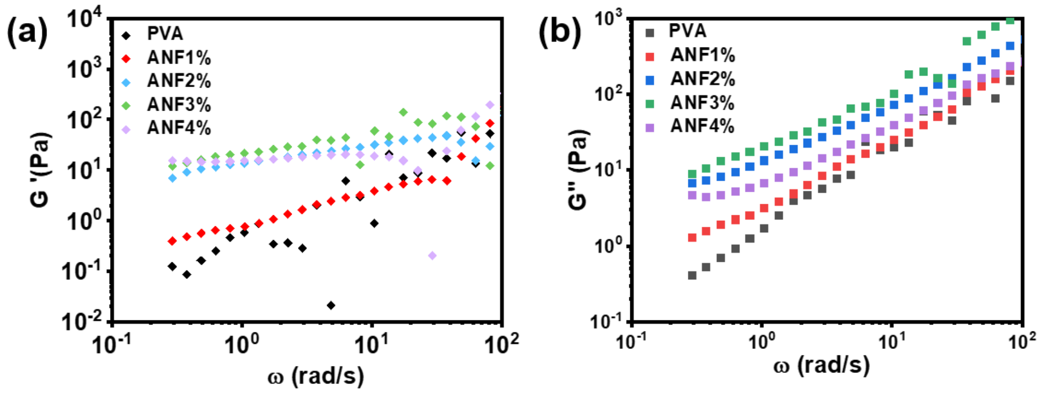

Figure S5. Frequency-dependent (a) storage modulus, and (b) loss modulus of ANF-PVA solution with different ANF contents.

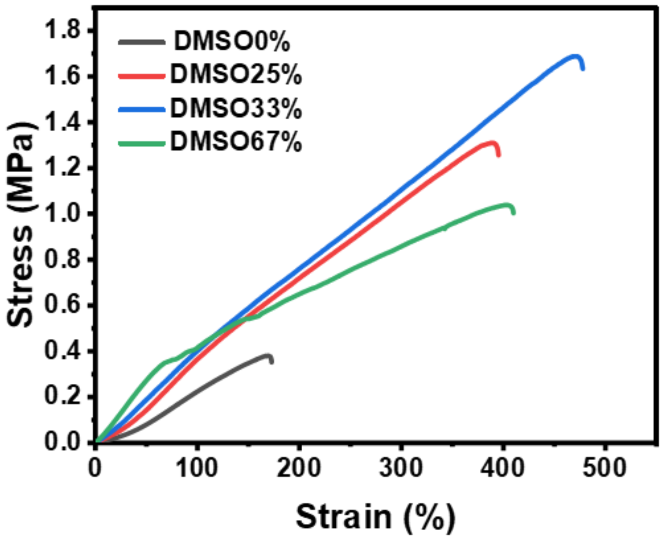

Figure S6. The stress versus strain curves of ANF-PVA organohydrogels containing 3 wt% ANFs with different DMSO contents.

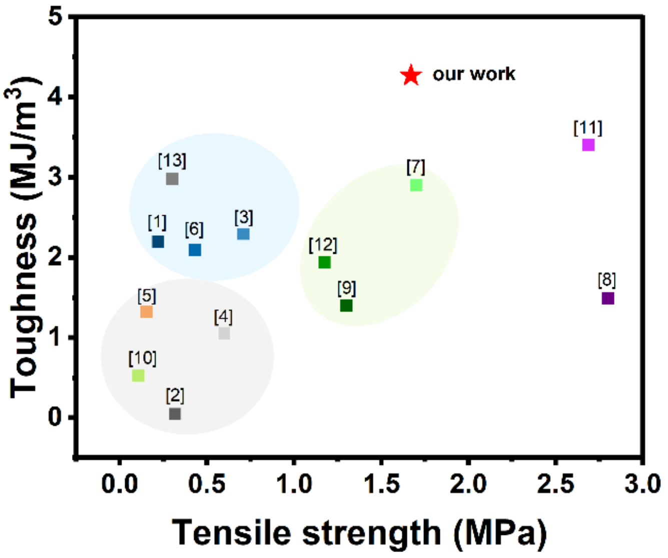

Figure S7. Comparison of the mechanical properties between the as-prepared ANF-PVA organohydrogels and previously reported hydrogels. [1-13]

Table S1. Initial freezing temperature of ANF-PVA organohydrogels with different ANF and DMSO contents.

Sample	DMSO33% ANF1%	DMSO33% ANF2%	DMSO33% ANF3%	DMSO33% ANF4%	DMSO0% ANF3%	DMSO25% ANF3%	DMSO67% ANF3%
**Initial freezing temperature (℃)**	-48	-47	-49	-45	-13	-34	/

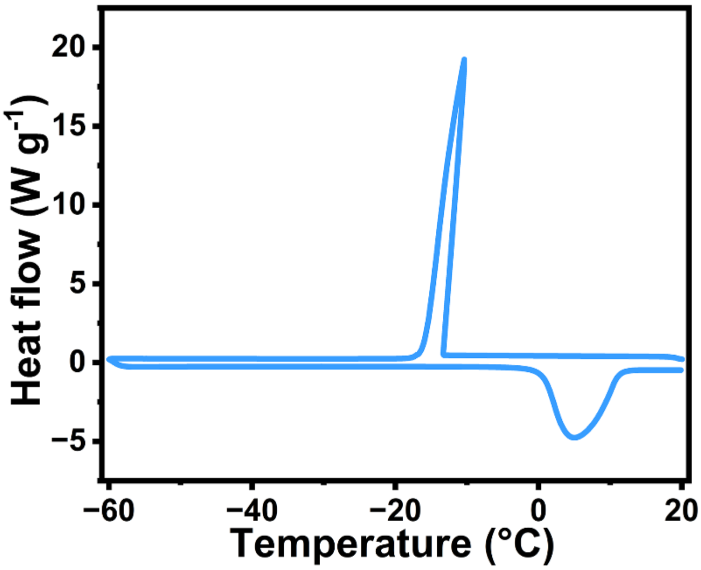

Figure S8. DSC curves for ANF3%-PVA hydrogel.

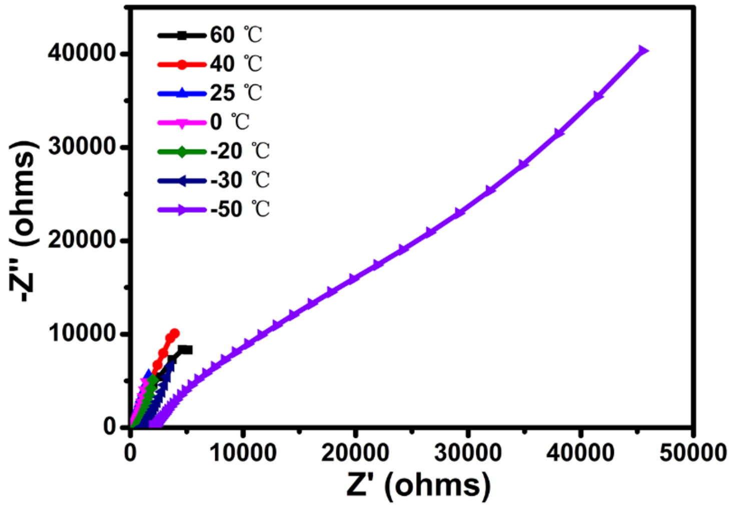

Figure S9. Nyquist plot of the organohydrogel electrolyte containing 6 M KOH at varied temperature.

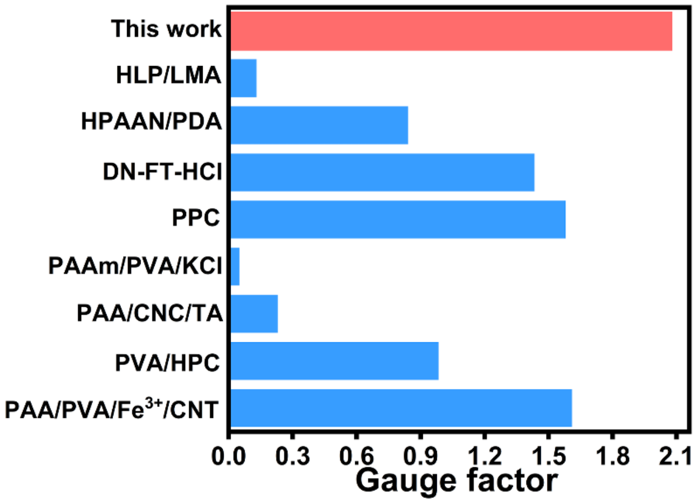

Figure S10. Summary of recent hydrogel-based flexible sensors.[14-21]

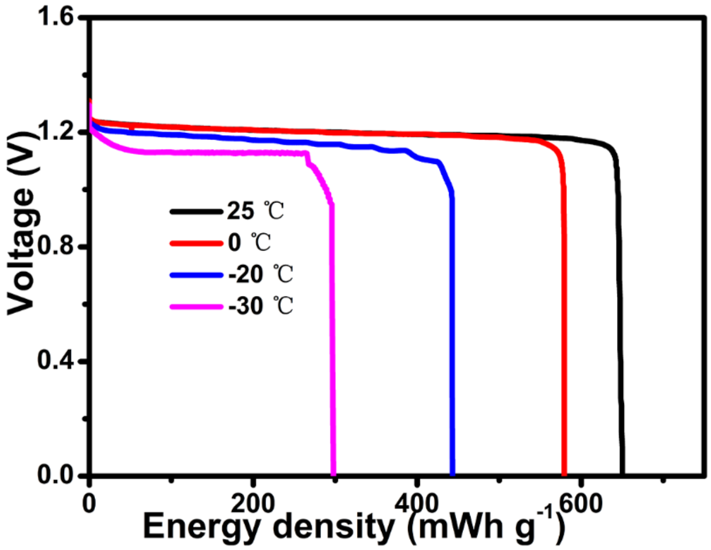

Figure S11. Energy density of ZABs in 25 ℃ to -30 ℃ environments at a current density of 2 mA cm^{-2}.

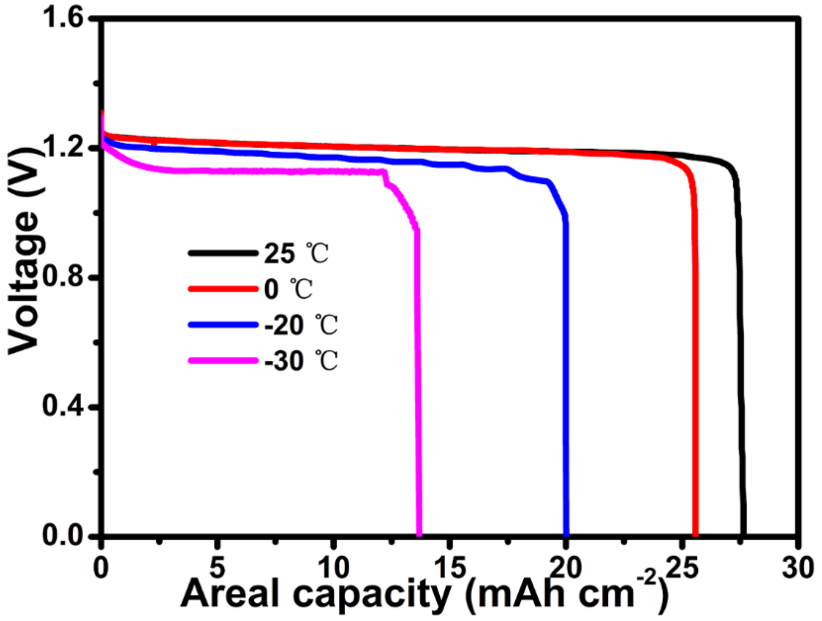

Figure S12. Areal capacity of ZABs in 25 ℃ to -30 ℃ environments at a current density of 2 mA cm^{-2}.

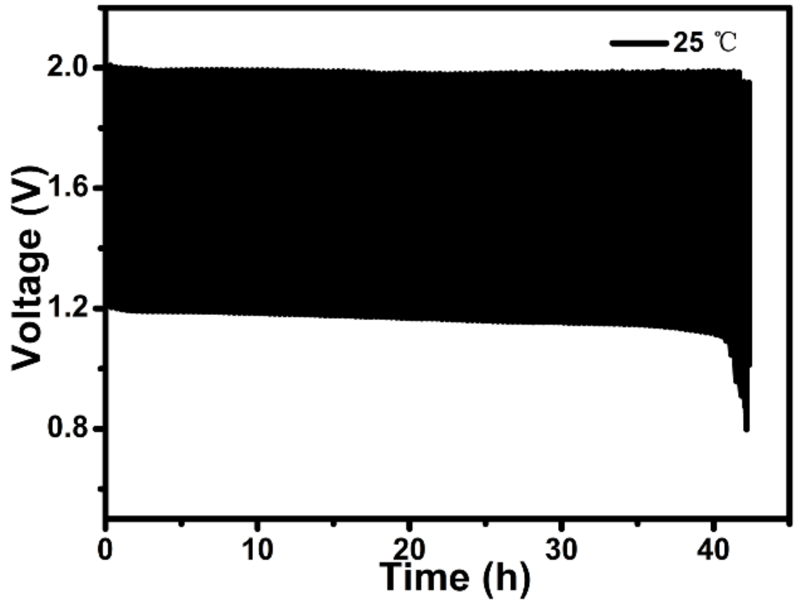

Figure S13. Cycling stability test of the OHE-based ZAB at a current density of 1 mA cm^{-2} at 25 ℃

.

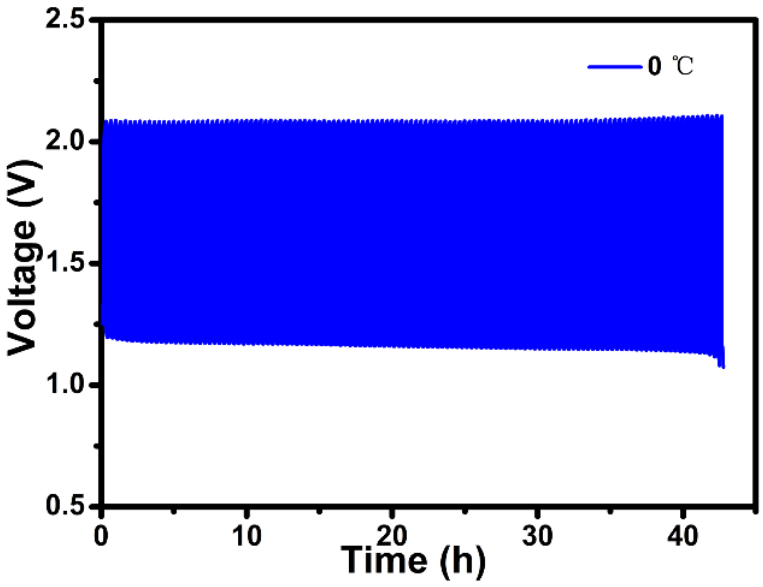

Figure S14. Cycling stability test of the OHE-based ZAB at a current density of 1 mA cm^{-2} at 0℃.

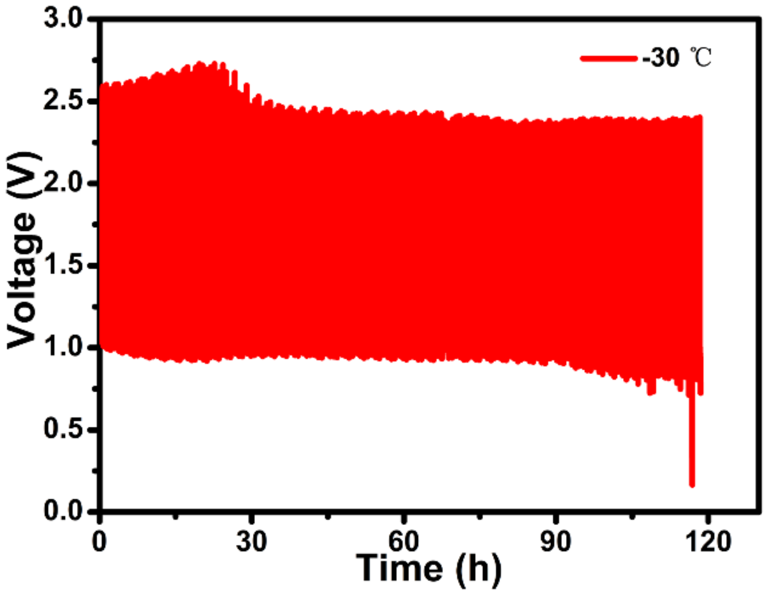

Figure S15. Cycling stability test of the OHE-based ZAB at a current density of 1 mA cm^{-2} at -30℃

.

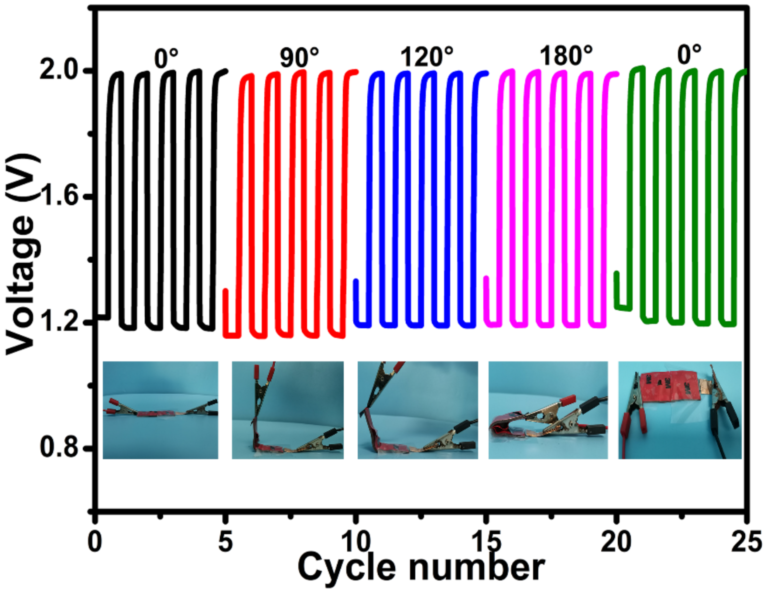

Figure S16. Cycling stability of the ZAB under different deformations at 25 °C.

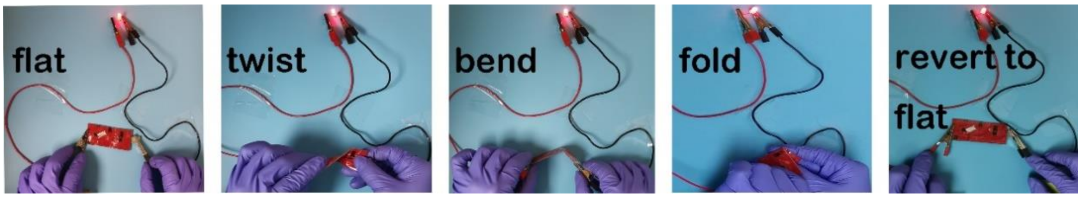

Figure S17. Demonstrations of OHE-based ZABs as power supply for LED bulb under different deformations.

Reference

[1] B. Yang, W. Yuan, *ACS Appl. Mater. Interfaces* 2019, **11**, 16765.

[2] Y. Yue, X. Wang, Q. Wu, J. Han, J. Jiang, *J. Colloid Interf. Sci.* 2020, **564**, 99.

[3] X. Sun, Z. Qin, L. Ye, H. Zhang, Q. Yu, X. Wu, J. Li, F. Yao, *Chem. Eng. J.* 2020, **382**, 122832.

[4] Y. Huang, M. Zhang, W. Ruan, *J. Mater. Chem. A* 2014, **2**, 10508.

[5] X. Su, S. Mahalingam, M. Edirisinghe, B. Chen, *ACS Appl. Mater. Interfaces* 2017, **9**, 22223.

[6] H. Ding, X. Liang, Q. Wang, M. Wang, Z. Li, G. Sun, *Carbohyd. Polym.* 2020, **248**, 116797.

[7] Y. Yang, X. Zhao, J. Yu, X. Chen, X. Chen, C. Cui, J. Zhang, Q. Zhang, Y. Zhang, S. Wang, Y.

Cheng, *ACS Appl. Mater. Interfaces* 2020, **12**, 34161.

[8] P. Wei, L. Wang, F. Xie, J. Cai, *Chem. Eng. J.* 2022, **431**, 133964.

[9] S. Choi, Y. Choi, J. Kim, *Adv. Funct. Mater.* 2019, **29**, 1904342.

[10] X. Su, B. Chen, *Carbohyd. Polym.* 2018, **197**, 497.

[11] R. Yang, C. Fan, Y. Dou, X. Zhang, Z. Xu, Q. Zhang, Y. Sun, Q. Yang, W. Liu, *Appl. Mater. Today* 2021, **24**, 101089.

[12] L. Fan, J. Xie, Y. Zheng, D. Wei, D. Yao, J. Zhang, T. Zhang, *ACS Appl. Mater. Interfaces* 2020, **12**, 22225.

[13] X. Lu, Y. Si, S. Zhang, J. Yu, B. Ding, *Adv. Funct. Mater.* 2021, **31**, 2103117.

[14] G. Ge, W. Yuan, W. Zhao, Y. Lu, Y. Zhang, W. Wang, P. Chen, W. Huang, W. Si, X. Dong, *J. Mater. Chem. A* 2019, **7**, 5949.

[15] Y. Zhou, C. Wan, Y. Yang, H. Yang, S. Wang, Z. Dai, K. Ji, H. Jiang, X. Chen, Y. Long, *Adv. Funct. Mater.* 2019, **29**, 1806220.

[16] C. Shao, M. Wang, L. Meng, H. Chang, B. Wang, F. Xu, J. Yang, P. Wan, *Chem. Mater.* 2018, **30**, 3110.

[17] G. Ge, Y. Zhang, J. Shao, W. Wang, W. Si, W. Huang, X. Dong, *Adv. Funct. Mater.* 2018, **28**, 1802576.

[18] R. Liu, K. Chen, H. Liu, Y. Liu, R. Cong, J. Guo, Y. Tian, *ACS Appl. Mater. Interfaces* 2022, **14**, 51341.

[19] J. Ren, Y. Liu, Z. Wang, S. Chen, Y. Ma, H. Wei, S. Lü, *Adv. Funct. Mater.* 2021, **32**, 2107404.

[20] Z. Gao, L. Kong, R. Jin, X. Liu, W. Hu, G. Gao, *J. Mater. Chem. C* 2020, **8**, 11119.

[21] S. Xia, Q. Zhang, S. Song, L. Duan, G. Gao, *Chem. Mater.* 2019, **31**, 9522.
